# Supplementary material for: Association between Antiviral Prophylaxis and Cytomegalovirus and Epstein–Barr Virus DNAemia in Pediatric Recipients of Allogeneic Hematopoietic Stem Cell Transplant
Source: Vaccines (Basel). 2021 Jun 7;9(6):610. doi: 10.3390/vaccines9060610 (PMC8226807; doi:10.3390/vaccines9060610)
Supplement: Supplementary file 1 [file vaccines-09-00610-s001.zip › vaccines-1206483-supplementary.pdf]

Table S1. Details of EBV and CMV qPCR tests according to study site

Site	qPCR method	Type of specimen	qPCR detection limits
**EBV DNA detection**			
CHU Sainte-Justine (Montreal)	In-house, Taqman	Whole blood	Baseline = 200 copies /mL.<br>Quantifiable range = 10^2-10^6 copies/mL.
CancerCare Manitoba (Winnipeg)	In house, Taqman	Whole blood	Lower detection limit = 500 copies/mL.<br>Quantifiable range = $5.75x10^2$ – $5.75x10^6$ copies/mL.
Alberta Children's Hospital (Calgary)	Commercial qPCR from Altona Diagnostics (Hamburg, Germany)	Whole blood	Lower detection limit = 289 IU/mL. Upper limit = $5x10^9$ IU/mL.<br>In the range 289 – 550 IU/mL these are reported as non-quantifiable <550 IU/mL.
BC Children's Hospital (Vancouver)	In house, Taqman, titered against WHO standard	Serum	Lower detection limit = 200 copies /mL.<br>Quantifiable range = $2x10^2$ - $4x10^6$ copies/mL.
**CMV DNA detection**			
CHU Sainte-Justine (Montreal)	In-house Taqman	Whole blood	Baseline = 200 copies /mL.<br>Quantifiable range = 10^2-10^6 copies/mL.
CancerCare Manitoba (Winnipeg)	In-house Taqman	Plasma	Quantifiable range = $2.75x10^2$ – $2.75x10^6$ copies/mL. Beyond this range, specimens are reported as < 275 copies/mL or >$2.75x10^6$ copies/mL.
Alberta Children's Hospital (Calgary)	Altona Diagnostics Real Star CMV PCR kit v1	Plasma	Lower detection limit = 41 IU/mL.<br>Dynamic range = 150 - $1.21x10^{12}$ IU/mL.<br>Non-quantifiable range: >41 to < 150 IU/mL.
BC Children's Hospital (Vancouver)	In-house Taqman, tittered against WHO standard	Serum	Specimens are reported positive at Ct < 40.<br>Quantifiable range = $3x10^3$ – $3x10^6$ copies/mL

Table S2. Characteristics of hematopoietic stem cell transplant (HSCT) recipients according to acyclovir and famciclovir use.

Variables		Acyclovir+	Acyclovir -	Famciclovir +	Famciclovir -
Number of patients (n)		n=117	n=39	n=43	n=113
EBV, n (%)	No	73 (62.39)	30 (76.92)	29 (67.44)	74 (65.49)
	Yes	44 (37.61)	9 (23.08)	14 (32.56)	39 (34.51)
CMV, n (%)	No	96 (82.05)	29 (74.36)	36 (83.72)	89 (78.76)
	Yes	21 (17.95)	10 (25.64)	7 (16.28)	24 (21.24)
Sex, n (%)	Male	59 (50.43)	24 (61.54)	24 (55.81)	59 (52.21)
	Female	58 (49.57)	15 (38.46)	19 (44.19)	54 (47.79)
Recipient age at transplant (years)	Mean (SD)	7.89 (5.50)	5.43 (4.35)	9.20 (4.97)	6.53 (5.30)
	Median (IQR)	6.63 (2.85-11.19)	4.57 (1.54-9.11)	7.81 (4.64-14.43)	5.2 (1.91-10.25)
Primary diagnosis, n (%)	Malignant	55 (47.01)	14 (35.90)	21 (48.84)	48 (42.48)
	No-malignant	62 (52.99)	25 (64.10)	22 (51.26)	65 (57.52)
Recipient pre-transplant EBV serology, n (%)	Negative	20 (17.1)	22 (56.41)	6 (13.95)	36 (31.86)
	Positive	86 (73.50)	15 (38.46)	35 (81.40)	66 (58.41)
	Unknow	11 (9.40)	2 (5.13)	2 (4.65)	11 (9.73)
Graft EBV serostatus, n (%)	Negative	45 (38.46)	17 (43.59)	19 (44.19)	43 (38.05)
	Positive	47 (40.17)	16 (41.03)	20 (46.51)	43 (38.05)
	Unknow	25 (21.37)	6 (15.38)	4 (9.30)	27 (23.89)
Donor match, n (%)	Donor matched	37 (31.62)	16 (41.03)	18 (41.86)	35 (30.97)
	Alternative donor	80 (68.38)	23 (58.97)	25 (58.14)	78 (69.03)
Graft source, n (%)	CB	29 (24.79)	10 (25.64)	7 (16.28)	32 (28.32)
	BM/PBSC	88 (75.21)	29 (74.36)	36 (83.72)	81 (71.68)
Conditioning regimen, n (%)	Other	75 (64.10)	23 (58.97)	36 (83.72)	62 (54.87)
	MAC	42 (35.90)	16 (41.03)	7 (16.28)	51 (45.13)
GvHD, n (%)	No	72 (61.54)	25 (64.10)	27 (62.79)	70 (61.95)
	Yes	45 (38.46)	14 (35.90)	16 (37.21)	43 (38.05)
Antithymocyte globulin, n (%)	No	72 (61.74)	20 (51.28)	27 (62.79)	65 (57.52)
	Yes	45 (38.46)	19 (48.72)	16 (37.21)	48 (42.48)
Alemtuzumab, n (%)	No	88 (75.21)	30 (76.92)	34 (79.07)	84 (74.34)
	Yes	29 (24.79)	9 (23.08)	9 (20.93)	29 (25.66)
Tacrolimus or CsA, n (%)	No	50 (42.74)	9 (23.08)	8 (18.60)	51 (45.13)
	Yes	67 (57.26)	30 (76.92)	35 (81.40)	62 (54.87)
MTX, n (%)	No	65 (55.56)	27 (69.23)	23 (53.49)	69 (61.06)
	Yes	52 (44.44)	12 (30.77)	20 (46.51)	44 (38.94)
MMF, n (%)	No	75 (64.10)	25 (64.10)	24 (55.81)	76 (67.26)
	Yes	42 (35.90)	14 (35.90)	19 (44.19)	37 (32.74)

ATG: antithymocyte globulin; BM: bone marrow; CB: cord blood; CsA: cyclosporine A; CMV: cytomegalovirus; EBV: Epstein-Barr virus; GvHD: Graft-versus-host disease; HHV: human herpesvirus; HSCT: hematopoietic stem cell transplant; IQR: interquartile range; MAC: myeloablative conditioning; MMF: mycophenolate mofetil; MTX: methotrexate; NA: not applicable; PBSC: peripheral blood stem cells; SD: standard deviation.
